# Genetic mutations in *SPINK1*, *CFTR*, *CTRC* genes in acute pancreatitis

**DOI:** 10.1186/s12876-015-0302-6

**Published:** 2015-06-23

**Authors:** Dorota Koziel, Stanislaw Gluszek, Artur Kowalik, Malgorzata Chlopek, Liliana Pieciak

**Affiliations:** 1 Faculty of Health Sciences, Jan Kochanowski University, Kielce, Poland; 2 Clinic General Oncological and Endocrinological Surgery, Regional Hospital, Kielce, Poland; 3 Department of Molecular Diagnostics, Holy Cross Cancer Centre, Kielce, Poland

**Keywords:** Acute pancreatitis, Etiology, *SPINK1* mutations, *CFTR* mutations, *CTRC* mutations

## Abstract

**Background:**

Explanation of the ultimate causes of acute and chronic pancreatitis is challenging. Hence, it is necessary to seek various etiological factors, including genetic mutations that may be of importance in triggering recurrence and progression of acute to chronic pancreatitis. The aim of this study was to determine the frequency of genetic mutations in patients with acute pancreatitis and to investigate their relationship with the etiology and clinical course.

**Methods:**

The study included 221 patients treated for acute pancreatitis and 345 healthy subjects as a control group. Peripheral blood samples were collected from each study participant and genomic DNA was isolated. Genotyping of common mutations in the SPINK1 (p.N34S and p.P55S) and CTRC (p.I259V, p.V235I, p.K247_R254del, p.E225A) genes was performed using the high-resolution melting method. Mutations in the CFTR p.F508del (delF508_CTT) were genotyped using allele-specific amplification polymerase chain reaction. All detected mutations were confirmed with direct capillary DNA sequencing.

**Results:**

Mutations in SPINK 1, CFTR and *CTRC* were detected in 6.3 %, 2.3 % and 1.8 % of patients with acute pancreatitis versus 3.2 %, 3.8 % and 1.2 % of volunteers in the control group. No relationship was found between the detected mutations and severity of acute pancreatitis: mild acute pancreatitis, mutation of *CFTR* in 4 (2.8 %) and *CTRC* in 2 (1.4 %) patients; severe acute pancreatitis, mutation of *CFTR* and *CTRC* in 1 (2.6 %) case each. The *SPINK1* mutation was significantly more frequent in 8 (10.4 %) severe cases than in 6 (4.2 %) mild cases (*P* < 0.05), and was observed in 5/70 (7.1 %) patients with alcohol-related AP, 5/81 (6.2 %) with biliary AP, and 4/63 (6.3 %) in those without any established cause of the disease.

**Conclusions:**

Mutation p.N34S in *SPINK1* may predispose patients to acute pancreatitis, especially in those abusing alcohol, and may promote a more severe course of the disease.

## Background

The most frequent causes of acute pancreatitis (AP) are gallstones and alcohol [1]. However, in a considerable number of individuals, even many attacks of gallstones and multiple episodes of alcohol abuse do not lead to acute pancreatitis [2, 3]. It was presumed that repeated acute pancreatitis and chronic pancreatitis, especially with a family history of pancreatic disease, may have a genetic background. It is considered that genetic etiology is responsible for ~25 % of all cases of chronic pancreatitis; however, it should be emphasized that ~40 % of cases are considered to be idiopathic [4].

Epidemiological investigations have confirmed high morbidity due to acute pancreatitis among adult inhabitants of the Kielce Region in Poland [5]. In 2011, pancreatic events occurred for the first time in 79.7/100,000 inhabitants. In many patients, idiopathic pancreatitis was diagnosed. The study showed that the taking of family and environmental history, which could explain the causes of the disease in some cases, was frequently omitted in the analysis of etiology [5]. This inclined us to explore further the collected epidemiological data.

In the pathogenesis of pancreatitis, various groups of genetic mutations may play an important role. Mutations in the cationic trypsinogen (*PRSS1*), anionic trypsinogen (*PRSS2*), pancreatic secretory trypsin inhibitor (*SPINK1*), cystic fibrosis transmembrane conductance regulator (*CFTR*), chymotrypsinogen (*CTRC*), calcium-sensing receptor (*CASR*) and the protein claudin-2 (*CLDN2*) genes were found in different types of pancreatitis [6–9]. The first described was a mutation of the trypsinogen gene in exon 3 (p.R122H). In other studies, other mutations in this gene were also analyzed, and related with hereditary pancreatitis, such as p.N291, p.A16V, p.R122C and p.D22G [10, 11].

The majority of studies have focused on determining the importance of various mutations in the etiology of pancreatitis. In the case of confirmed presence of a mutation, the course of acute episodes of pancreatitis is rarely investigated in the context of various environmental factors and other factors known as determinants of acute pancreatitis.

The results of studies of mutations and their contribution to the etiology of acute and chronic pancreatitis are divergent; therefore, it seems justifiable to analyze further patients with acute pancreatitis, which is a life-threatening event, and remains an important clinical challenge.

The aim of this study was to determine the frequency of genetic mutations in *SPINK1*, *CFTR* and *CTRC* genes in patients with acute pancreatitis (AP), as well as to investigate their relation with the etiology and clinical course.

## Methods

The study included 221 patients with acute pancreatitis, inhabitants of the Kielce Region in Poland, who gave their informed consent for collecting genetic material. Diagnosis of mild, moderate or severe acute pancreatitis (SAP), according to the Atlanta 2012 classification, was the criterion for inclusion in the study. Diagnosis of AP was based on the joint interpretation of medical history, physical examination and targeted laboratory tests. The diagnosis was based upon satisfaction of at least 2 of the following 3 criteria: (1) upper abdominal pain of sudden onset, frequently radiating towards the back; (2) lipase or amylase activity in serum >3 times the upper limit of normal; and (3) results of imaging tests that allow one to obtain cross-sectional images: computed tomography (CT), nuclear magnetic resonance (NMR), or ultrasonography (USG). In the analysis of the etiology of AP, medical history taking can determine alcohol consumption, occurrence of concomitant diseases, drug intake, procedures performed, and endoscopic retrograde cholangiopancreatography (ERCP). Biliary etiology was confirmed based on USG, CT, NMR, and/or ERCP, only if there were indications for performance of these procedures, for example, icterus or triple elevation of alanine aminotransferase level. Endoscopic ultrasonography was performed in some patients to confirm biliary AP. Alcohol consumption was evaluated with the Short Alcohol Dependence Data (SADD) Questionnaire, and self-estimated alcohol consumption via interview. Acute pancreatitis was diagnosed if a patient gained ≥10 points on the SADD Questionnaire, or if a period of alcohol abuse lasted ≥1 year, and the daily dose was 40 g of pure ethanol.

Diagnosis of chronic pancreatitis was an exclusion criterion. The control group included adult volunteers selected by random sampling, without any apparent concomitant diseases that could have affected the structure and expression of genes to be tested in the study.

The control group consisted of 345 healthy inhabitants of the Kielce Region: 223 women and 122 men; mean age 45.1 (44 in women, 47 in men) years, in a general good state of health, with body mass index 18.5–30. The study group included 221 patients who had acute pancreatitis (88 female, 133 male); mean age 55.4 (56.2 in women, 51.4 in men). The course of AP was mild in 65.2 % of patients, moderately severe in 17.6 %, and severe in 17.2 %. The percentage of women in the study group was significantly lower compared with the control group, while the mean age was significantly higher in the study than control group.

The study was approved by the Committee on Bioethics at the Faculty of Health Sciences, Jan Kochanowski University in Kielce. Each patient and member of the control group gave their informed consent to perform the genetic testing.

### DNA isolation

Peripheral blood samples were drawn from all study participants and collected in EDTA tubes. DNA was isolated using Micro AX Blood Gravity Kit (A&A Biotechnology, Gdańsk, Poland).

Quality and concentration of isolated DNA was measured by Nano Drop 2000 (Thermo Scientific, TK Biotech, Poland).

Genotyping was performed using the following molecular biology techniques: allele-specific PCR (ASA-PCR) and high-resolution melting (HRM)-PCR, and all detected mutations were confirmed by direct capillary sequencing. To validate ASA-PCR and HRM-PCR, the first 164 samples were sequenced in parallel, irrespective of genotyping results.

### Mutation detection in *CFTR* gene

The p.F508del (delF508_CTT) mutation was detected using 10 μl ASA-PCR mixture containing 5 μlSybr Green Kit (Qiagen, Syngen-Biotech, Wrocław, Poland), 1 μl water, 1 μl each primer, and 1 μl DNA template. PCR was performed in a Veriti 96 Well Thermal Cycler (Applied Biosystems, USA). The cycling profile was set at 95 °C initial denaturation for 1 min, followed by 35 cycles of 15 s at 95 °C, 15 s at 60 °C, 30 s at 72 °C, and a final extension at 72 °C for 7 min. ASA-PCR product detection and visualization were performed using a microchip electrophoresis system MCE-202 Multi NA (Shimadzu, Shim-pol, Warsaw, Poland).

### Mutation detection in *SPINK1* and *CTRC* genes

Mutation detection was performed using primers (Table 1) flanking genetic regions containing the following mutations in: *SPINK1* exon 3 (p.N34S and p.P55S), and *CTRC* exon 3 and 7 (p.I259V, p.V235I, p.K247_R254del, and p.E225A).

**Table 1:** Sequences of primers used for the reaction and methods used

Gene	Method		Primer sequence
CFTR ex10	allele-specific PCR	F R M	GCAAGTGAATCCTGAGCGTG
			TGGGTAGTGTGAAGGGTTCAT
			GCACCATTAAAGAAAATATCATTGG
SPINK1 ex3	HRM/capillary sequencing	F R	TTGCTATGAACTCAAGAATGGAGA
			CCGATTTTCAAAACATAACACTG
CTRC ex7	HRM/capillary sequencing	F R	CTTATGCCCTCCCGGTCTGG
			GGACAGCTGTGGAGGCAG
CTRC ex3	HRM/capillary sequencing	F R	CTGACACACAGCCCTCCC
			ATGGCCAGGTCTCAGGGTAT

HRM-PCR mixture was composed of 5 μl Type-it HRM PCR Kit (Qiagen, Synge-Biotech), 7 μl water, 1 μl each forward and reverse primer (Table 1), and 1 μl template DNA. PCR was performed by using Rotor Gene (Qiagen, Syngen-Biotech). Cycling conditions were: 5 min initial denaturation at 95 °C, followed by 40 cycles of 10 s at 95 °C, 30 s at 65 °C, 30 s at 65 °C, but during the first 10 cycles, we used a touchdown mode of 1 °C per cycle, 10 s at 72 °C, and a heteroduplex formation consisting of 10 s at 95 °C and 20 s at 40 °C. Melting temperature range was 75–80 °C for exon 3 *SPINK1*, and 75–95 °C for exon 7 *CTRC*. For *CTRC* exon 3, there were 37 cycles with touchdown (1 °C/cycle) and a melting range of 80–93 °C.

Polymorphisms and mutations in the HRM-PCR products were detected based on a change in the shape of a PCR product melting curve. All detected polymorphisms and mutations were then confirmed by capillary sequencing.

For capillary sequencing, the primers used – HRM-PCR products (Table 1) – were enzymatically purified using Exo SAP reagent containing phosphatase and exonuclease. The purified PCR product was sequenced with BigDye Terminator v3.1 Cycle Sequencing Kit (Applied Biosystems). The reaction conditions were 25 cycles of 96 °C for 10 s, 55 °C for 5 s, and 60 °C for 105 s). The reaction products were purified after sequencing by using Big Dye X Terminator Kit (Applied Biosystems). Sequencing was carried out on a 3130 Genetic Analyzer (Applied Biosystems). The resulting chromatograms were analyzed manually and compared using BLAST software from the NCBI site (Table 1).

### Statistical analysis

Differences between the 2 groups were analyzed by examining the proportions of patients in each group representing a particular category (e.g., occurrence of *SPINK1* mutation), using the 1-tailed Z test for comparing 2 proportions.

The relationships between gender and occurrence of *SPINK1*, *CFTR* or *CTRC* mutations in patients with AP and in the control group were analyzed using a more conservative test. The data sets were displayed in the form of 2 × 2 contingency tables with gender as the row variable, and occurrence of mutation as the column variable. Some cell numbers were small and there were big differences between cell numbers. The null hypothesis that the specific mutation occurred equally in men and women was verified with Fisher’s exact test. Odds ratios wereto compare the odds of occurrence of a specific event in the study group versus the control group, and 95 % confidence intervals were also reported.

## Results

There were no significant differences in frequencies of *SPINK1*, *CFTR* and *CTRC* mutations when comparing the male and female patients. The data sets were displayed in the form of 2 × 2 contingency tables with gender as the row variable and occurrence of mutations as the column variable. Some cell numbers were small and large differences occurred between cell numbers. In such a case, Fisher’s exact test was applied to analyze the relationships between the variables. The null hypothesis was that the specific mutation occurred equally in men and women (Table 2).

**Table 2:** Contingency Table for analysis of the association between gender and mutation in *SPINK1, CFTR, CTRC* in the group of patients with AP and the control group of healthy individuals without a past history of acute pancreatitis

	*SPINK 1*			*CFTR*			*CTRC*		
	Yes	No	*p*-value	Yes	No	*p*-value	Yes	No	*p*-value
Patients with AP									
Female	5	83	NS^*^	4	84	NS	1	87	NS
Male	9	124		1	132		3	130	
*Marginal Column Totals*	14	207	-	5	216	-	4	217	-
Control group of healthy individuals									
Female	8	215	NS	6	217	NS	2	221	NS
Male	3	119		7	115		2	120	
Marginal Column Totals	11	334	-	13	332		4	341	-

NS^*^ no statistical significance

In patients with AP, the gene mutations detected compared with the controls were as follows: *SPINK1*, 6.3 % patients versus 3.2 % controls; *CFTR*, 2.3 % patients versus 3.8 % controls; and *CTRC*, 1.8 % patients versus 1.2 % controls (Table 3).

**Table 3:** Clinical course of AP according to causes of the disease in the group of patients with mutation diagnosed

	Alcohol	Gallstones	Cancer	Idiopathic	Control population
	*N* = 70 (31.7 %)	*N* = 81 (36.7 %)	*N* = 7 (3.2 %)	*N* = 63 (28.5 %)	*N* = 345
*SPINK1 n* = 14 (6.3 %)					11 (3.2 %)
Mild AP	2 (2.9 %)	2 (2.5 %)	0	2 (3.2 %)	
Moderate and severe AP	3 (4.3 %)	3 (3.7 %)	0	2 (3.2 %)	
*CFTR n* = 5 (2.3 %)					13 (3.8 %)
Mild AP	1 (1.4 %)	2 (2.5 %)	1 (14.3 %)	0	
Moderate and severe AP		1 (1.2 %)	0	0	
*CTRC n* = 4 (1.8 %)					4 (1.2 %)
Mild AP	1 (1.4 %)	1 (1.2 %)	0	0	
Moderate and severe AP	1 (1.4 %)	1 (1.2 %)	0	0	

HRM-PCR and direct sequencing detected 12 p.N32S and 2 p.P55S mis-sense mutations in *SPINK* in patients, which together constituted 6.3 % of AP cases. However, using ASA-PCR, p.F508del mutations were found in 5 (2.3 %) AP cases. HRM-PCR and direct sequencing detected 4 (1.8 %) mutations in *CTRC*. All detected mutations were of the mis-sense type, that is, 2 p.V235I, 1 p.I259V and 1 p.K247_R254del (Table 4).

**Table 4:** Mutations detected in the *SPINK 1, CFTR, CTRC* genes in correlation with clinical data

Gene mutation	No. of patients	BMI	Alcohol abuse (≥40 g daily)	Cigarette smoking (currently)	Pancreatic diseases in family	Concomitant diseases	Recurrences
*SPINK1* p.N34S c.101A > G exon 3	12 (5.42 %)						
1		26.0	Yes	No	No	None	Yes
2		20.2	Yes	No	No	None	No
3		29.8	No	No		None	No
4		24.0	No	No	No	cardiovascular diseases, atherosclerosis, Parkinson’s disease	No
5		29.4	No	No	No	None	No
6		23.4	Yes	Yes	No	None	Yes
7		22.4	Yes	Yes	No	gallstones, gastroesophageal reflux disease, psychoorganic syndrome	No
8		27.7	No	No	No	None	No
9		26.4	No	Yes	Yes	arterial hypertension	Yes
10		25.4	No	No	No	None	Yes
11		30.1	Yes	Yes	No	arterial hypertension, type-2 diabetes	No
12		26.6	Yes	No	No	None	No
*SPINK1* p.P55S c.163C>	2 (0.9 %)						
1		27.0	Yes	No	No	None	
2		19.0	No	No	No	cardiovascular diseases, gallstones	No
*CFTR*p.F508del (delF508) c.1521_1523 delCTT C > T exon 3	5 (2.3 %)						
1		20.2	No	No	Yes	cardiovascular diseases	No
2		26.4	No	No	No	arterial hypertension	Yes
3		25.6	No	No	No	arterial hypertension	No
4		19.2	No	No	No	pancreatic cancer	No
5		26.0	Yes	Yes	No	None	No
*CTRC* p.V235I c.703G > A exon 7	2 (0.9 %)						
1		23.4	Yes	Yes	No	None	No
2		22.9	Yes	Yes	No	None	Yes
p.I259V c.775A > C exon 7	1 (0.5 %)						
1		27.0	No	No	Yes		Yes
p.K247_R254 delc.738_761del24 exon 7	1 (0.5 %)						
1		26.2	No	No	No	None	No

In 1 patient with recurrence of moderately severe AP with alcohol etiology, we observed p.N34S mutation in *SPINK1* and p.V235I mutation in *CTRC*.

### *SPINK1*

The *SPINK1* mutation was more common in the AP than the control group. The observed proportions were 0.063 and 0.032, respectively. The Z score was 1.777, and the corresponding *P* value was 0.03. The patients with mild AP had a significantly lower proportion of mutations in *SPINK1* than those with moderately severe or severe AP. The observed proportions were 0.042 and 0.104, respectively. The Z score was 1.8095 and the *P* value was 0.03.

In considering the causes of AP (alcoholic, biliary and idiopathic), no differences were found in the frequency of mutation in *SPINK1*. No significant differences were observed between the frequency of mutations in *SPINK1* in the subgroups with and without recurrence.

The study group without recurrent AP had a higher proportion of mutation in *SPINK1* than the control group had, and the difference was almost significant at the 0.05 level. The observed proportions were: 0.065 and 0.032, respectively. The Z score was 1.6436 and the *P* value was 0.0505 (Table 5).

**Table 5:** Frequency and odds ratios (95 % CI) of mutations in *SPINK1, CFTR, CTRC* in the individual groups with acute pancreatitis and in the control group

	No.	SPINK1* 14/221 (6.3 %)		OR * (95 % CI)	*P value*	CFTR** 5/221 (2.3 %)	*OR (95 % CI)*	*P value*	CTRC** 4/221 (1.8 %)		*OR (95 % CI)*	*P value*
Severity of the course												
Mild AP	144	6 (4.2 %)		2.05 (0.85-5.1)	*P* < 0.005	4 (2.8 %)	0.59 (0.16- 1.80)	NS	2 (1.4 %)		1.57 (0.29-8.52)	NS
Moderate AP	39	7 (17.9 %)	8 (10.4 %)			0			2 (1.4 %)	1,4 %		
Severe AP	38	1 (2.6 %)				1 (2.6 %)			1 (2.6 %)			
Etiology of AP												
Alcohol	70	5 (7.1 %)		2.33 (0.61- 7.57)	NS	1 (1.4 %)	0.37 (0.01-2.55)	NS	2 (2.9 %)		2.50 (0.22- 17.84)	NS
Gallstones	81	5 (6.2 %)		1.99 (0.53- 6.45)		3 (3.7 %)	0.98 (0.18- 3.7)		2 (2.5 %)		2.15 (0.19-15.33)	
Idiopathic	63	4 (6.3)		2.05 (0.46- 7.23)		0	0 (0–1.78)		0		0 (0–8.365)	
Cancer	7	0		0 (0–23.88)		1 (14.3 %)	4.2 (20.09- 39.08)		0		0 (0–85.65)	
Reccurrences of AP												
Yes	82	5 (6.1 %)		1.97 (0.52- 6.37)	NS	1 (1.2 %)	0.32 (0.01- 2.16)	NS	2 (2.4 %)		2.13 (0.19- 15.135)	NS
No	139	9 (6.5 %)		2.10 (0.75- 5.72)		4 (2.9 %)	0.76 (0.18- 2.51)		2 (1.4 %)		1.24 (0.111-8.8	
Control group	345	11 (3.2 %)*				13 (3.8 %)**			4 (1.2 %)**			

Indicates that the odds of SPINK1 mutations are 2.05 times higher in the AP group than in the control group. The limits (0.847; 5.096) for OR 95 % confidence interval are not very wide, so the OR estimate has rather good precision. The association between the group and the SPINK1mutations occurrence is not significant at the 0.05 level because 95 % confidence interval for the OR contains 1. However, right-sided F exact test rejects the null hypothesis that OR = 1 in favour of the alternative hypothesis that true odds ratio is higher than 1 (*p* = 0.0466). This means that the odds of SPINK1 in the AP group are higher than in the control group, and this conclusion is statistically significant at the 0.05 level

NS no statistical significance

*SPINK1 mutation in the acute pancreatitis group (AP) is more frequent than in the control group: *p* < 0.005

**The differences between the *CFTR*, *CTRC* mutations in the acute pancreatitis group (AP) and in the control group are insignificant

### *CFTR*

There was no significant difference in the frequency of mutation in *CFTR* in the AP and control groups. The observed proportions were: 0.023 and 0.038, respectively. The Z score was −0.9959 and the *P* value was 0.15 (Table 5).

### *CTRC*

There was no significant difference in the frequency of mutation in *CTRC* in the AP and control groups. The observed proportions were: 0.018 and 0.012, respectively. The Z score was 0.6396 and the *P* value was 0.26 (Table 5).

## Discussion

SPINK1 is a specific trypsin inhibitor and an acute phase protein that is secreted by the acinar cells. SPINK1 protein plays a role in the prevention of premature activation of zymogen that is catalyzed by trypsin within the pancreatic duct system or the acinar tissue. A reactive site in the protein serves as a specific target substrate for trypsin. *SPINK1* polymorphisms are common in the general population (~2 %) and are significantly associated with pancreatitis [12]. The results of studies of p.N34S *SPINK1* mutation in acute pancreatitis are divergent. Tukiainen et al. [13] evaluated the frequency of mutation in *SPINK1* in 371 patients with acute pancreatitis, including 207 with the mild form and 164 with the severe form. The control group comprised 459 individuals. The p.N34S mutation occurred in 29 patients (7.8 %) and 12 individuals (2.6 %) in the control group. In the majority of patients, the disease had an alcohol-related etiology (*n* = 229; 61 %). The frequency of p.N34S mutation was higher in the group with severe disease (15/164; 9.1 %) and with alcoholic etiology (21/229; 9.2 %). The differences were not statistically significant. No differences between age and number of attacks were observed in the groups examined. The researchers concluded that the p.N34S mutation in *SPINK1* may increase susceptibility to acute pancreatitis. O’Reilly et al. [14] confirmed the relationship between this mutation and AP. The above-mentioned mutation occurred significantly more often in patients with AP. The authors suggest that this mutation predisposes to premature protease activation in the development of pancreatitis. In turn, Aounet al. [15] found that p.N34S mutation in *SPINK1* was not associated with the first attack of pancreatitis; however, it increased the risk of recurrence. In the present study, the frequency of *SPINK1* mutations in 221 patients with pancreatitis was 6.3 %, which was significantly higher than in the control group (*P* = 0.03). This mutation also occurred significantly more frequently in the patients with moderate and severe forms of AP (*P* = 0.03), compared with those with a mild course of the disease. Our study confirmed the importance of this mutation as a predisposing factor for AP, and was simultaneously responsible for the more severe course of the disease. No differences were observed in the frequency of mutations in individual subgroups according to the cause (alcoholic, biliary, idiopathic, and others). Thus, our observations and those of other researchers suggest that *SPINK1* mutations predispose to severe pancreatitis. Such a relationship was not found in studies of Polish patients with CP. However, it should be stated that the group examined was several times smaller than that investigated in the present study [16].

The studies conducted to date suggest that the spectrum of mutations in *SPINK1* is geographically varied: the IVS 3 + 2 T > C mutation is more frequent in Chinese children with Idiopathic chronic pancreatitis (ICP), whereas p.N34S is more frequent among western populations. The role of IVS3 + 2 T > C mutation has not been finally recognized. It may exert an effect on gene transcription; or it may be the cause of translocation of the entire exon 3 where there is a binding site for trypsin, damaging the splicing donor site, and in the final phase, causing disorder in the protease/antiprotease balance in the pancreas. Further studies are necessary to explain the molecular mechanisms [17].

In the present study, the p.N34S mutation in *SPINK1* was most frequently observed (5.4 % in patients with AP and 0.3 % in the control group), and more rarely, p.P55S mutation (0.9 % and 0.6 % in the control group). The IVS3 + 2 T > C mutation in *SPINK1* often occurred in the study by Wanga et al. [11] – 57.3 % of cases; however, they did not find any p.N34S mutation in *SPINK1*. Kume et al. [18] observed these mutations in 13–17 % of Japanese patients. The IVS3 + 2 T > C mutation in *SPINK1* was often noted in Korean patients with idiopathic and familial pancreatitis [19]. Studies conducted in western countries indicated that this mutation occurred in only 1 (1 %) of 96 patients, and in 3 (2.7 %) of 112 pediatric patients with ICP [20]. Pfutzer et al. [21] showed that N34S mutation in *SPINK1* was seen in 40 % (23/57) of American children with ICP, while Truninger et al. [10] found it in 43 % (6/14) of German patients with early onset of ICP.

Wang et al. [11] observed that the frequency of mutation in *SPINK1* (57.3 %) was higher than that described by Witt et al. (19–40 %). Mystakidis et al. [22] did not find any mutation in *SPINK1* in a group of 30 patients with pancreatitis after ERCP, and concluded that this mutation does not play any important role in the pathogenesis of this type of pancreatitis. Nevertheless, we consider that more comprehensive studies should be carried out to confirm these results. In our study, no patients developed post-ERCP pancreatitis.

Recent studies have suggested an important role for CFTR in the development of pancreatitis, particularly through its role in intraluminal pH regulation from bicarbonate secretion and the flushing of ductal proteins. Secretory granules of pancreatic acinar cells co-release protons with digestive enzymes during normal pancreatic secretion. Diminished ductal bicarbonate secretion and consequent reduced alkalinization of the acinar lumen may promote the development of pancreatitis since acidification of the pancreatic lumen can lead to a loss of tight junction integrity, allowing the leakage of digestive enzymes into the pancreatic duct lumen and interstitial space [23]. Stressors, such as oxidative damage, overloading the protein-folding capacity of the endoplasmic reticulum, trigger the unfolded protein response [24].

Obstructive tubulopathy of the pancreas is the result of *CFTR* dysfunction and plays a primary role in the development of CP; however, the precise process of the course of the inflammation has not been fully established. The function of *CFTR* in the pancreas is important for the dilution and alkalinization of protein-rich secretions, and therefore, plays a preventive role in the formation of protein plugs in the pancreatic ducts. Partial loss of *CFTR* function may be related to idiopathic and alcohol-related pancreatitis. Recently, Schneider et al. [25] have described that the co-occurrence of R75Q mutation in *CFTR* and variants of *SPINK1* was considerably higher in patients with ICP, compared with the control group (8.75 % vs. 0.38 %). Using the patchclamp technique, they found that *CFTR* may be the cause of disturbed secretion of bicarbonates and increased risk of pancreatitis. A large number of *CFTR* mutations and the presence of specific *CFTR* genotype are associated with pancreatitis. In the present study, there was no significant difference in the proportions of mutations in *CFTR* in the AP group (5/221; 2.3 %) and control group (13/345; 3.8 %). The observed proportions were: 0.023 and 0.038, respectively. The Z score was −0.9959 and the *P* value was 0.15866. No relationship was confirmed in mutations with severe AP: mild AP mutation of *CFTR* in 4 (2.8 %) patients, and in the SAP mutation of *CFTR* in 1 (2.6 %) patient. Rosendahl et al. [26] examined 660 patients with chronic pancreatitis and noted that the accumulation of *CFTR* variants in chronic pancreatitis was lower than described previously; nevertheless, the presence of these variants may have increased the risk of development of the disease by 2.7–4.5-fold. The studies confirmed a complex genetic interaction with CP, and a smaller effect of *CFTR* variants in the development of the disease.

The mechanisms by which *CTRC* protects against pancreatitis have been established; however, the importance of *CTRC* variants in terms of risk for RAP and CP is less clear [27]. The *CTRC* mutations may predispose to CP through ineffective trypsin degradation, ineffective carboxypeptidase activation, and induction of endoplasmic reticulum [27]. The *CTRC* mutation is related with chronic pancreatitis in European and Asian populations. Wang et al. [11] did not find mutations in Chinese children with idiopathic pancreatitis, and indicated that *CTRC* mutation may vary geographically and ethnically. In our own studies, *CTRC* mutation occurred with a similar frequency in the study group with AP (4/221; 1.8 %) and in the control group (4/345; 1.2 %). No differences were observed between individual subgroups of AP severity. No p.A73T mutations in *CTRC* were found in the patient or control group. The p.K247_R254del mutation in *CTRC* was confirmed in 1 patient with AP and 3 healthy individuals. The remaining mutations examined, p.E225A, p.V235I and p.I259V in *CTRC*, occurred singly in the group of patients with AP and in the healthy volunteers.

Chang et al. [28] evaluated a Chinese cohort of cases of CP and associated additional mutations with CP, but did not replicate the previous findings. Rosendahl et al. [26] showed that the rare p.R254W and p.K247_R254del variants were significantly over-represented in cases of pancreatitis in Germany.

## Conclusions

The N34S mutation of *SPINK1* may predispose to AP, especially in patients who abuse alcohol, and may result in a more severe course of the disease. The accumulation of various mutations and environmental factors may be of importance in the development of AP.

## Abbreviations

AP: Acute pancreatitis
CP: Chronic pancreatitis
*PRSS1*: Cationic trypsinogen gene
*PRSS2*: Anionic tripsinogen
*SPINK1*: The pancreatic secretory trypsin inhibitor gene
*CFTR*: The cystic fibrosis transmembrane conductance regulator gene
*CTRC*: The chymotrypsinogen gene
*CASR*: The calcium-sensing receptor gene
*CLDN2*: The protein claudin-2
SAP: Severe acute pancreatitis
CT: Images computed tomography
NMR: Nuclear magnetic resonance
USG: Ultrasonography
ERCP: Endoscopic retrograde cholangiopancreatography
HRM: High resolution melting
ASA-PCR: Allele-specific PCR
ICP: Idiopathic chronic pancreatitis.
